# Dural plasmacytoma mimicking meningioma in a young patient with multiple myeloma

**DOI:** 10.2349/biij.5.2.e5

**Published:** 2009-04-01

**Authors:** NN Rahmah, HK Brotoarianto, E Andor, G Kusnarto, Z Muttaqin, K Hongo

**Affiliations:** 1 Department of Neurosurgery, Shinshu University School of Medicine, Matsumoto, Japan; 2 Department of Neurosurgery, Diponegoro University, Dr. Kariadi Hospital, Semarang, Indonesia

**Keywords:** multiple myeloma, posterior fossa, intracranial plasmacytoma

## Abstract

Intracranial involvement in multiple myeloma (MM) is rarely found, especially with dural involvement. There are only a few cases found concerning MM with intracranial involvement. MM usually involves an older group of patients. Cases involving young patients are very rare. The differential diagnosis of a dural plasmacytoma includes meningioma, metastasis, lymphoma and sarcoma of the dura mater. We present a young patient, 33 years old, with MM presenting an intracerebral mass mimicking meningioma on MRI. MM was diagnosed the previous year. The patient presented with headache, balance disturbance and back pain. MRI revealed an occipital extra-axial mass with a dural tail. Histopathological examination after excision showed MM. Published literatures on intracranial involvement of MM are also discussed. Plasmacytoma should be considered in the differential diagnosis of a solitary dural mass, particularly in a patient with MM.

## INTRODUCTION

Multiple myeloma (MM) is a malignant tumor that may involve the vertebrae and cranium. In the skull, they are typically multiple lytic lesions, exhibiting a ‘moth-eaten’ appearance on radiograph [1]. The term plasmacytoma refers to a single lytic lesion of malignant plasma cells infiltration. From a classical point of view; intracranial plasmacytomas and MM may involve the cranial vault and/or the skull base only, the brain parenchyma, arising in the cranial vault and/or the skull base and the orbit [2]. However, intracranial involvement in plasmacytomas and MM is rare [2]. In this study, an intracranial mass of dural origin in a patient with one year history of MM is reported. Review of the literatures is also presented.

## CASE REPORT

**Figure 1:**
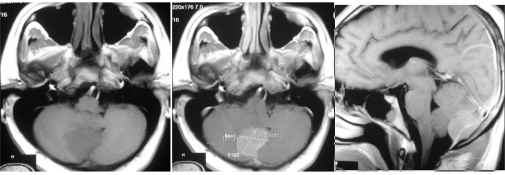
MRI study demonstrated a homogenously enhanced intracranial mass of the posterior fossa. (left) hypointense mass on T1-weighted image, (centre) well-absorbed contrast agent on T1 with contrast agent, (right) note the hyperintense line in the middle of the mass of the sagittal view.

A 33-year-old male was referred to the neurosurgical department by an internist with chronic headache. MRI study (0.5 T) performed revealed a posterior fossa mass. The mass was located behind the cerebellum, 31.3 x 31.2 x 23.0 mm in size and enhances post contrast (Figure 1). It has similar appearance to meningioma, but with a past history of MM, intracranial MM was considered as a differential diagnosis.

**Figure 2:**
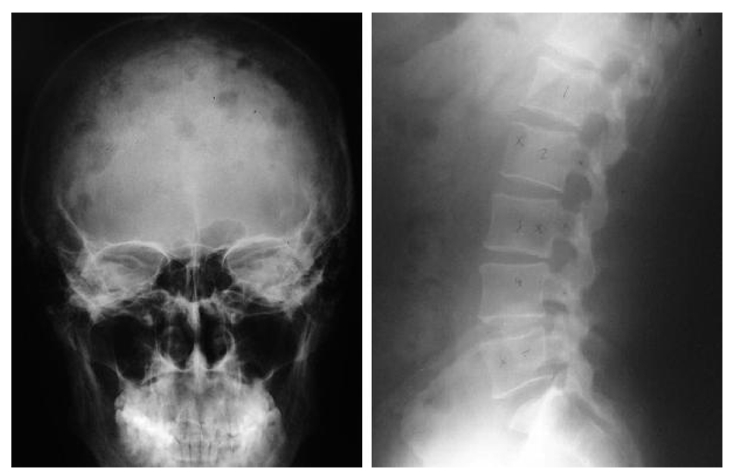
Series of X-photo revealed osteolytic lesions. (left) AP view of the skull, (right) lateral view of the vertebra.

The earlier diagnosis of MM was established by chance. The patient complained of back pain a year ago and underwent several examinations including plain radiographs. Several lytic lesions of the bones were noticed (Figure 2). Laboratory examinations performed, were positive for Bence Jones protein. At the time of referral, he had already started chemotherapy of capecitabine.

He underwent surgery for the posterior fossa lesion. Preoperatively, the patient was fully alert and showed no neurological deficit. Suboccipital approach craniotomy was performed. Skin was incised vertically, 5 cm in length, in the midline between the external occipital protuberance and C_2_ process. Bone was drilled just below the external occipital protuberance and was removed so that foramen magnum was widened.

The mass was visualized under the occipital bone, and it was found to have infiltrated the bone. The mass was grayish, soft on palpation and easily bled. After being cauterized from its surrounding blood supply, the extradural mass was removed and a small round defect was seen on the dura. The dura was then incised T-shaped and an intradural mass was visualized. Its appearance was similar to the extradural mass, and it had to be carefully cauterized before its removal from the surrounding structures. Arachnoid layer was perfectly attached and total mass removal was done. Dura was closed by dural plasty using fascia and the bone defect was left open considering that muscles in that area were thick enough to replace its function.

**Figure 3:**
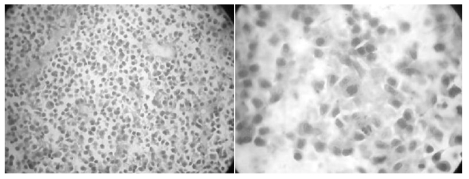
Histopathological study of the intracranial mass, (left) monomorphous spread of plasma cell (40x), (right) round-oval cell with eccentric nuclei and abundant cytoplasm, and increased nuclei cytoplasm ratio, and mitotic phase of plasma cell.

Postoperatively, the patient was alert and showed no additional neurological deficit. Histopathological study with H&E staining demonstrated a monomorphous appearance of plasma cells with characteristic of round-oval cells, eccentric nuclei, and abundant cytoplasm. There was also an increased nuclear to cytoplasmic ratio and some cells had prominent nucleoli (Figure 3). It confirmed a diagnosis of plasma cells tumor. Unfortunately, patient died within one year after the diagnosis of intracranial involvement of MM.

## DISCUSSION

MM is a neoplasm of a single clone of plasma cells. It is characterized by proliferation of plasma cells in the bone marrow, infiltration of adjacent tissues with mature and immature plasma cells, and the production of immunoglobulin, usually monoclonal IgG or IgA (referred to collectively as M protein) [3]. Plasmacytoma is referred to a single lesion without any evidence of MM in any other part of the body [1,4]. Primary extramedullary plasmacytomas are uncommon, accounting for 4% of all plasma cell tumors, mainly arising in the head and neck, particularly the upper aerodigestive tract. Plasmacytomas generally present as bone or soft-tissue tumors with a variable mass effect, pain, and infiltrative behavior [2]. The typical clinical features of MM are bone pain, weakness, fatigue, fever and infection.

Chiang et al in his 11 plasmacytoma-case report stated that solitary plasmacytoma arises from medullary tissue, and is believed to be an early manifestation of MM [5]. Within 3 to 5 years, approximately 50% of these tumors eventually disseminate throughout the skeletal system and become indistinguishable from MM. Patients are most commonly diagnosed during the fifth to sixth decade of life, and 70% are men [5]. Schwartz et al reported intramedullary plasmacytoma progressed to MM more frequently (approximately 50%) than extramedullary plasmacytomas (approximately 30%). Intracranial, calvarial lesions and most cranial base lesions, being intramedullary, also have a greater chance of progressing to MM than do dural-based lesions [6]. Nevertheless, one review of the literature demonstrated that, of 18 calvarial and 13 dural-based lesions, only 2 calvarial lesions progressed to MM [7]. In contrast, Bindal et al., who presented two cranial base plasmacytomas and reviewed the literature on intracranial plasmacytomas, observed that all cranial base-infiltrating plasmacytomas were associated with MM [8]. Extracranially, MM may take several years to develop from a plasmacytoma [6].

Interestingly from previous literature, several reports were made on MM. Some mentioned the progress of solitary plasmacytoma into MM; others mentioned case reports of patients with MM and metastasized into intracranial lesions, whereas several reports did not put clear borders between the two. This patient presented to the authors because of a history of MM; therefore, the authors would like to find published reports in the literatures with similar appearance. By excluding those who were admitted with intracranial plasmacytoma as their first reason for admission, and excluding those who had no MRI and/or autopsy report confirming the intracranial involvement of MM, 24 cases were found [2,6,9-23]. Details were shown in table 1. Mean age was 56.2 years old, with female predominance. Mean interval before diagnosis of intracranial involvement was established was 20.57 months. 90% of cases died within a month after diagnosis was established. 34% of those cases were IgG kappa MM, and 30.4% were IgG alpha. Petersen et al reported on 54 cases of MM with myelomatous meningeal involvement and a male/female ratio of 2:4. This type of involvement occurred more frequently in IgA- and IgD-MM and in Durie-Salmon Stage III disease. They suggested that advanced stage of MM observed in cases with circulating plasma cells in the peripheral blood showed a hematogenous spread of tumor cells to the meninges [20].

**Table 1:** Characteristics of Multiple Myeloma Patients with Intracranial Involvement

**Author**	**Cases**	**Age/Sex**	**MM type**	**Lytic lesion**	**Tx**	**Intracranial location**	**Interval (m)**	**Tx**	**Diagnosis**	**Outcome**	**COD**
Ben-Basset (1968)	1	69/F	IgD	NR	NR	meninges	NR	NR	autopsy	dead in 16w	NR
Maldonado (1970)	1	40/F	NR	NR	NR	meninges	NR	n	autopsy	dead in 1m	increased ICP
McCarthy (1978)	1	49/M	IgG	n	cx	meninges frontoparietal	12	intrathecal cx	autopsy	dead in 2w	progression
Oda K (1990)	1	64/F	IgG K, IIIB	y	cx	meninges diffuse	36	craniospinal rx	LP, autopsy	dead in 4m	pneumonia
Leifer D (1992)	1	67/F	IgG A	n	cx,radiation	CN 3,5,7,sup cereb peduncle, ffrontal	24	intrathecal cx	MRI,CSF	dead in 2w	stroke
Maulopoulos (1993)	2	48/F	NR	y	cx	meninges occipital	NR	NR	MRI,CSF	dead in 9m	progression
		63/M	NR	n	cx	meninges parietal	NR	resection	histology	NR	NR
Hirata (1996)	1	43/F	IgG K	y	cx	meninges diffuse	12	intratechal cx	MRI,CSF	dead in 1m	pneumonia
Turhal N (1998)	1	27/M	IgG A	n	cx	sphenoclival	12	radiaton,cx	MRI,CSF	dead	sepsis
Petersen SL (1999)	1	39/M	IgG A	y	cx	meninges	12	ntrathecal cx	MRI,CSF	dead in 9d	progression
Roddie P (2000)	1	55/F	IgG K	y	cx	meninges,cavernous sinus	12	cx	MRI	NR	NR
Schwartz (2001)	2	49/M	IgG K	NR	radiotherapy	NR	26	refused	histology	dead	progression
		54/F	IgG A	NR	radiotherapy	NR	48	n	histology	dead	progression
Sahin F (2004)	1	50/F	IgG K	y	cx,radiation	meninges temporal	84	resection, rx	histology	dead	progression
Montalban (2005)	1	54/M	IgG A	y	n	sphenoclival	6	cx	MRI,histology	dead in 9m	progression
Haegelen C (2006)	1	72/F	IgG K	n	mass resection	meninges	3	cx	MRI	dead in 9m	progression
Jablonski (2006)	1	58/?	NR	NR	NR	meninges temporal	12	NR	MRI	NR	NR
Tsang CS (2006)	1	61/M	IgG A	y	NR	meninges frontoparietal	NR	biopsy	CT, histology	dead in 1w	progression
Gozetti A (2007)	2	62/F	IgA K IIIA	n	cx	cavernous sinus	9	cx	MRI	dead in 8m	sepsis
		80/F	nonsecret IIIA	n	cx	cavernous sinus	8	cx	MRI	dead (soon)	progression
Husein OF (2007)	1	54/F	NR	NR	cx,stem cell	petrous apex,clivus	48	biopsy, rx	histology	good in 18m	progression
Cerase A (2008)	3	61/F	IgG K IIIA	NR	cx,stem cell	cavernous sinus,clivus	9	NR	MRI	NR	NR
		79/F	nonsecret IIIA	NR	cx	cavernous sinus,clivus	8	palliative rx	MRI	dead (soon)	progression
		73/F	IgG A	NR	cx (resistant)	meninges	10	NR	MRI	dead (soon)	progression
**Our case**	1	33/M	NR	y	cx	meninges occipital	12	resection	MRI,histology	dead in 1y	progression
**Total**	**25**										

Tx:therapy, COD: cause of death, F:female, M:Male, NR:not reported, n:none, CN:cranial nerve, cx:chemotherapy, rx:radiotherapy, LP:lumbar puncture, CSF:cerebrospinal fluid

52.2% of cases involved the cranial vault, and 47.8% were in skull base. Of those, only 1 case involved the posterior fossa dura. Intracranial plasmacytoma involving the posterior fossa in a young patient such as this patient is a rare case. The authors found only 24 cases of MM with intracranial involvement in the literatures, and most of them were beyond 40 years of age. Ishida et al reported a total of 32 cases of MM in patients aged 30 years and below in the literatures [24]. The authors' patient died within 1 year after being diagnosed, and most of the cases found in the literatures also showed short survival rates. Higurashi et al reported prediction indicators of progression to MM that were of intramedullary nature, the existence of residual tumor, and location in the skull base [25]. 60-70% of intramedullary lesions arising from the bone progress to MM, compared with 10% to 20% of extramedullary lesions arising from the soft tissues, such as the mucosal linings of the middle ear, mastoid air cells, and paranasal sinuses. Many of the skull base lesions that were initially considered to be solitary eventually progressed to MM [25]. They suggested that most plasma cell myelomas of the skull base are of intramedullary nature and are actually in the early stage of MM. On the contrary, plasma cell myelomas of the other cranial locations are mostly of extramedullary nature and appear to be solitary plasmacytoma [25].

MRI study in this patient revealed a single oval lesion in the posterior fossa, which was enhanced homogenously by contrast injection, had defined border with cerebellum, and was located under the occipital bone. It appeared similar to meningioma; in addition, the lesion showed an enhanced line dividing the mass in half which was thought to be a blood supply to the meningioma. Plasmacytoma can be mistaken with meningioma since it has typical radiological features as an extra-axial mass with broad attachment to the dura, hyperdense on CT, isointense with grey matter on T1-weighted MRI, more variable signal intensity on T2-weighted MRI, and strongly and homogeneously enhancing with contrast [2,4,11,13,26]. During operation, it was revealed that the mass was located extra- and intradural, and that enhanced line in the middle of the lesion was actually the dura itself. Sahin et al found that intracranial plasmacytomas are usually highly vascular and pathological vessels and a tumor blush may be seen on angiography. However, the majority of dural or intraparenchymal plasmacytomas are less vascular and angiography may be normal. Therefore, angiography is not reliable for differential diagnosis, as 30-50% of meningiomas may also be associated with normal angiography [18]. Radiological findings of intracranial MM or plasmacytoma are not specific. They may mimic lymphoma, metastasis, sarcoma of the dura mater, osteochondroma, infectious meningitis or meningioma [27,28]. The authors also examined the mass histopathologically. The mass revealed monomorphous plasma cells, which were in accordance with his history of MM. Dural origin mass and bone-attached mass were both examined. Both pointed out the same type of cells.

In conclusion, despite its systemic manifestation, MM may appear as intracranial mass. Although dural infiltration is very rare, it should be considered in the differential diagnosis, particularly if a solitary intracranial mass with typical features of meningioma is detected on MRI. Its origin and MRI appearance should be carefully observed before performing surgery.
